# The Question is Still Open: Is Supplemental Oxygen Enhancing Performance in Professional Athletes at High Altitude or Not?

**DOI:** 10.1007/s40279-022-01803-y

**Published:** 2023-01-31

**Authors:** Nikolaus C. Netzer, Martin Faulhaber, Hannes Gatterer, Tobias Dünnwald, Wolfgang Schobersberger, Kingman P. Strohl, Stephan Pramsohler

**Affiliations:** 1 Hermann Buhl Institute for Hypoxia and Sleep Research, Department of Sport Science, University of Innsbruck, Fürstenweg 185, 6020 Innsbruck, Austria; 2 Department of Sport Science, University of Innsbruck, Innsbruck, Austria; 3 Division of Sports Medicine and Rehabilitation, University Hospital Ulm, Ulm, Germany; 4 Present Address: Institute for Mountain Emergency Medicine, Eurac Research, Via Hypatia 2, 39100 Bozen, Italy; 5 Department of Psychology and Sportsmedicine, University for Health Sciences, Medical Informatics and Technology, Innsbruck, Austria; 6 Department of Medicine, University Hospitals, Case Western Reserve University, Cleveland, OH USA

Dear Editor,

Skiing Birds of Prey in Beaver Creek as a recreational skier may be challenging. You can see people stop after a few yards of monster moguls and gasp for air. Drug stores in the valley sell tourists canned supplemental oxygen to cope with the “high altitude” at Beaver Creek Ski Resort. The yearly Super G or giant slalom events at a starting altitude of 3483 m also challenge professionals and a shortage of oxygen is said to potentially influence their performance.

In the giant slalom 2018, the German racer Stefan Luitz breathed supplemental oxygen before the race and between legs, aiming to mitigate the hypoxia-related detrimental effects. In the end, he won the race, yet was stripped of his medal and World Cup points after the FIS (International Ski Federation) decided by its rules that the supplemental oxygen was against doping rules. The German team argued that the World Anti-Doping Agency had excluded supplemental oxygen from its doping list because research could not show a substantial enhancement with oxygen in performance, except during the competition itself. The Germans appealed the FIS decision to the Court of Arbitration for Sport and won [1]. Stefan Luitz was given his win back and prize money and is now officially listed in the winners list.

The Court of Arbitration for Sport and the World Anti-Doping Agency most likely referred in their decision to several publications concluding that supplemental oxygen does not enhance several different types of muscular work. A recent publication regarding skiing at altitude and supplemental oxygen was designed and executed with members of our own working group as co-authors [2]. Tobias Dünnwald and colleagues gave individuals 100% oxygen versus hypoxic air for 15 min during recovery periods between five extreme flywheel sets of maximal exercise with eccentric and concentric movement performed at a simulated altitude of 3500 m. Concentric and eccentric peak performance were not different between groups. In addition, when 100% oxygen is given for 5 min before a single peak maximal performance of 60 s at a 3500-m simulated altitude, there was no difference in peak performance between oxygenated and ambient air [3]. Similar to peak performance, another study showed that breathing hyperoxic air at a simulated altitude of 3500 m before a balance test does not enhance balance [4]. Balance is next to peak muscle performance as one of the most important abilities for a successful ski race.

Previous research conducted in cross-country skiers at intermediate altitude supports the findings of the Innsbruck research group [5, 6].

However, to be fair, there are those that propose that supplemental oxygen increases the performance of athletes at moderate-to-high altitudes. There are publications which report a positive effect of oxygen on performance at high altitude during training and competition [7, 8].

Although a higher muscle tissue oxygenation could not be found with breathing hyperoxic air between exercise intervals, it appeared to reduce the decline in power during maximal exercise in a hypoxic environment [9]. Second, muscles and muscle performance are only one part of an athletically perfect race. Next to psychological mood, the cognitive functions of the brain, sensory reaction time, and the recognition and assessment of obstacles probably play an important role for an individual to win a race. Actual research shows how observations can be made on the function of the brain via an electroencephalogram in real and virtual ski races [10]. Although still speculative, future research might show how the recognition of an obstacle is associated with an elevated high alpha power in the electroencephalogram of a skier. Like muscles, some, but not all, parts of the brain increase their oxygen consumption substantially, in part to metabolize lactate as an energy substrate [11]. However, the brain will not react to a low oxygen supply exactly like the muscles.

As we were able to show, particularly if a challenging performance happens after a night at high altitude, motor reaction time is not substantially impaired but sensory reaction time is [12]. The reduced processing speed is especially observed in the first 24 h at altitude [13], which is important for an athlete at a ski World Cup. After more time, the brain may start to return towards better functioning.

Based on existing research on muscle performance, the World Anti-Doping Agency rules and regulations seem to be adequate. However, it could be that these regulations have to be reconsidered based on experiments addressing overall human performance and define and/or include an effect on brain function. Thus, we consider the question of what oxygen does for performance at high altitude as still open.

## Data Availability

Not applicable.

## References

[1] Alpine skiing: anti-doping. Court of arbitration in sport media release upholds. The appeal of Stefan Luitz. 15 March 2019. https://www.tas-cas.org/medias/liste-de-diffusion/cas-media-release-alpine-skiing-the-appeal-of-stefan-luitz-isupheld.html. Accessed 19 Jan 2023.

[2] Dünnwald T, Morawetz D, Faulhaber M, Gatterer H, Birklbauer C, Koller A (2021). Supplemental O_2_ during recovery does not improve repeated maximal concentric-eccentric strength-endurance performance in hypoxia. J Strength Cond Res.

[3] Morawetz D, Dünnwald T, Faulhaber M, Gatterer H, Höllrigl L, Raschner C (2019). Can hyperoxic preconditioning in normobaric hypoxia (3500 m) improve all-out exercise performance in highly skilled skiers? A randomized crossover study. Int J Sports Physiol Perform..

[4] Morawetz D, Dünnwald T, Faulhaber M, Gatterer H, Schobersberger W (2019). Impact of hyperoxic preconditioning in normobaric hypoxia (3500 m) on balance ability in highly skilled skiers: a randomized, crossover study. Int J Sports Physiol Perform.

[5] Wilber RL, Holm PL, Morris DM, Dallam GM, Subudhi AW, Murray DM (2004). Effect of FIO_2_ on oxidative stress during interval training at moderate altitude. Med Sci Sports Exerc.

[6] Hauser A, Zinner C, Born DP, Wehrlin JP, Sperlich B (2014). Does hyperoxic recovery during cross-country skiing team sprints enhance performance?. Med Sci Sports Exerc.

[7] Wilber RL, Holm PL, Morris DM, Dallam GM, Callan SD (2003). Effect of F(I)O(2) on physiological responses and cycling performance at moderate altitude. Med Sci Sports Exerc.

[8] Morris DM, Kearney JT, Burke ER (2000). The effects of breathing supplemental oxygen during altitude training on cycling performance. J Sci Med Sport.

[9] Zinner C, Hauser A, Born D-P, Wehrlin JP, Holmberg H-C, Sperlich B (2015). Influence of hypoxic interval training and hyperoxic recovery on muscle activation and oxygenation in connection with double-poling exercise. PLoS ONE.

[10] Petukhov IV, Glazyrin AE, Gorokhov AV, Steshina LA, Tanryverdiev IO (2020). Being present in a real or virtual world: a EEG study. Int J Med Inform.

[11] Ide K, Secher NH (2000). Cerebral blood flow and metabolism during exercise. Prog Neurobiol.

[12] Pramsohler S, Wimmer S, Kopp M, Gatterer H, Faulhaber M, Burtscher M (2017). Normobaric hypoxia overnight impairs cognitive reaction time. BMC Neurosci.

[13] Falla M, Papagno C, Dal Cappello T, Vögele A, Hüfner K, Kim J (2021). A prospective evaluation of the acute effects of high altitude on cognitive and physiological functions in lowlanders. Front Physiol.

